# Editorial: Signaling in the Phytomicrobiome

**DOI:** 10.3389/fpls.2017.00611

**Published:** 2017-04-24

**Authors:** Donald L. Smith, Valérie Gravel, Etienne Yergeau

**Affiliations:** ^1^Plant Science Department, McGill UniversitySte. Anne de Bellevue, QC, Canada; ^2^Centre INRS-Institut Armand-Frappier, Institut National de la Recherche Scientifique, Université du QuébecLaval, QC, Canada

**Keywords:** molecular signals, plant growth promoting rhizobacteria, phytomicrobiome, holobiont, crop

Over the last decade we have come to appreciate that there are close relationships between all “higher” organisms and communities of microbes. The human microbiome and its role in human metabolism and health, is being widely investigated. In a similar way, plant-associated microbial communities are now coming under scrutiny.

Plants have probably had associated microbes since they colonized the land about 0.5 billion years ago. The terrestrial environment presented water and nutrient acquisition challenges resulting in the evolution of sophisticated plant root systems. However, associated microbes also help address these hurdles, and at lesser energetic costs (Smith et al.). Because most energy enters the terrestrial biosphere at the green leaves of plants, organisms associated with plants have advantageous access to reduced carbon from photosynthesis. So, when plants prosper, associated microorganisms benefit. Microbes are associated with all plant structures, but roots are in constant contact with generally humid, microbe-laden soil, and so are associated with the greatest number and range of microbes. The earliest evidence we have of plant-microbe interactions are fossils showing mycorrhizal relationships from almost 400 million years ago (Smith et al.).

We now realize that a plant growing under field conditions is community, not just an individual. While the circumstances of associated microbes are improved when the plants are doing well, the plants must at the same time control their associated microbes, to minimize the presence of those that are potentially detrimental. The microorganisms that colonize plants are collectively termed “the phytomicrobiome”. The genomes of the phytomicrobiome expand the genetic repertoire of the plant. This association has led to the redefinition of Karl August Möbius' biocenosis (metaorganisms comprising the macroscopic host and its synergistic interdependence with microbes) concept into the holobiont (an individual host and its microbial community) concept (Theis et al., 2016). The holobiont collective genome is the hologenome, the evolutionary unit; the phytomicrobiome is much more flexible than the plant genome and more readily modified than the hologenome (Nogales et al., 2016).

## Plant-phytomicrobiome signaling

It is becoming clear that plants exert control over the composition of their phytomicrobiome (Smith et al.). This is reviewed extensively in the recent Frontiers in Plant Science Research Topic “Signaling in the Phytomicrobiome.” Some of the regulatory activity by the plant is through availability of metabolites, but it is also increasingly evident that signals (exo-hormones or hormones of the holobiont) are being exchanged between the plant and members of its phytomicrobiome. Activities within the phytomicrobiome are also regulated through signaling, for instance through quorum sensing (Hartmann et al., 2014; Sitaraman; Smith et al.; Smith et al.), and other less well characterized signaling systems (e.g., Hagai et al., 2014).

Members of the phytomicrobiome can assist plant growth in a range of ways (Smith et al.). For instance, establishment of a specific phytomicrobiome on plants, such as willows, can allow them to better tolerate soil contamination, and so allow them to play a more effective role in phytoremediation (Bell et al., 2015; Yergeau et al., 2015). Some soil nutrients are relatively immobile (e.g., phosphorus and zinc) and some microbes, such as arbuscular mycorrhizal fungi (AMF), facilitate uptake of these nutrients by increasing effective root surface area; other microbes use chelators and other molecular interventions to help mobilize plant nutrients. Another key role of the phytomicrobiome is atmospheric nitrogen fixation. Indeed, nitrogen is the plant nutrient required in the greatest amounts; it is quite mobile in soils and it can become rapidly depleted.

The best understood example of signaling between a plant and elements of the phytomicrobiome occurs between leguminous plants and associated nitrogen-fixing rhizobia (Lira et al.; Nelson and Sadowsky; Smith et al.; Tóth and Stacey). Isoflavonoids secreted by plant roots guide rhizobial cells to the roots and activate key genes within the rhizobial cells, including the genes encoding production of lipo-chitooligosaccharides (LCOs) that signal back to the plant. Each legume species produces its own characteristic suite of isoflavonoids and it is generally the case that only the correct rhizobia respond to these. In a similar way, each type of rhizobia produces distinct LCOs, to which only the correct legume species responds (Smith et al.). The LCOs turn on a set of nodulation-related genes within the legume, initiating nodulation. In a few cases, the correct LCOs induce formation of completely differentiated nodules, in the absence of rhizobial cells. The presence of other phytomicrobiome members can enhance the nodulation process (Maymon et al.), although the mechanism is not understood. LCOs also serve as signals in the mycorrhizal relationship, suggesting that this is an ancient signaling system. However, the plant-to-mycorrhizal fungi signal is distinct from the plant-to-rhizobia signal, being strigolactone (Smith et al.), not an LCO and more related to the homoserine lactone used in quorum sensing among bacterial populations. Interestingly, rhizobia can also produce the plant growth promoting compound lumichrome, another phytomicrobome signal (Dakora et al.).

## Manipulating the phytomicrobiome

A better understanding of plant-microbiome signaling could help find novel ways to manipulate the microbiome to improve the plant holobiont's nutrition and resistance to stress. For instance, we have learned that the correct isoflavonoids can be added to rhizobial inoculants, to activate the nodulation genes prior to application onto plants (Smith et al.). This can overcome environmental stresses disrupting signal exchange and enhance the establishment of the nitrogen-fixing symbiosis. We have also learned that LCOs can stimulate plant growth directly, particularly under stressful conditions (Smith et al.; Subramanian et al., 2016a,b). Interestingly, it has been shown that jasmonate, a plant hormone which regulates plant responses to stressful conditions, can be excreted from plant roots and can activate genes that produce LCOs in some rhizobia; this has been shown to ameliorate plant response to stress (Smith et al.). Commercial products based on these understandings are now available for application to a range of crops (Smith et al.).

When one isolates bacteria from plant roots, *Bacillus* species are generally present. Recently, a strain of *Bacillus* which enhances plant growth under a range of conditions was isolated (Subramanian and Smith). This microbe was found to produce a small protein (thuricin 17) that, like the LCOs, stimulates plant growth at very low concentrations, and particularly when the plants are stressed. This protein is a bacteriocin that has a dual action by removing closely related competitors from the niche space, and promoting plant growth, thus enlarging the niche space, for the producing strain (Subramanian and Smith).

It is clear that the role of the phytomicrobiome is large, well developed and well-orchestrated. It is also clear that there is considerable potential in managing this system (Smith et al.; Quiza et al.) and that the use of “biologicals” will develop during the twenty first century and play as large a role as agro-chemistry did in the twentieth century. Biologicals can be deployed to enhance plant pathogen resistance (Ravichandran et al.). They can be used to enhance crop productivity, to meet the expanding demands for plant material as food, fiber and fuel. They can assist crop plants in dealing with the more frequent and more extreme episodes of stress that will occur as climate change conditions continue to develop. The path is clear and we have started down it; there is a considerable distance remaining.

## Author contributions

DS was the overall editor of the theme volume. EY and VG were junior editors of the theme volume and contributed to the writing of this editorial.

## Funding

Funding was provided for basic and applied research through the Natural Sciences and Engineering Research Council of Canada (grant number RGPIN-2015-06328) and from the Canadian Networks of Centres of Excellence (grant number G234970).

### Conflict of interest statement

The authors declare that the research was conducted in the absence of any commercial or financial relationships that could be construed as a potential conflict of interest.
